# Identifying prodromal biomarkers for schizophrenia and bipolar disorder using magnetoencephalography

**DOI:** 10.1192/j.eurpsy.2022.1641

**Published:** 2022-09-01

**Authors:** O. Jefsen, M. Dietz, K. Friston, O. Mors, Y. Shtyrov

**Affiliations:** 1 Aarhus University Hospital, Psychosis Research Unit, Aarhus C, Denmark; 2 Aarhus University, Center Of Functionally Integrative Neuroscience, Department Of Clinical Medicine, Aarhus, Denmark; 3 University College London, Wellcome Centre For Human Neuroimaging, London, United Kingdom

**Keywords:** bipolar disorder, Magnetoencephalography, familial high-risk, schizophrénia

## Abstract

**Introduction:**

Schizophrenia (SZ) and bipolar disorder (BD) are severe mental illnesses with large overlapping heritability. Both disorders are associated with altered neurophysiological responses, as measured with magnetoencephalography (MEG) or electroencephalography (EEG), particularly reduced mismatch negativity (MMN) and 40 Hz auditory steady-state responses (ASSR). These deficits could potentially both serve as early markers of illness and provide insight into the underlying pathophysiology as endophenotypes. First-degree relatives to patients with SZ and BD also show some neurophysiological deficits, however whether these deficits can be detected in adolescent offspring of patients is undetermined.

**Objectives:**

We aim to investigate whether adolescents at familial high risk of schizophrenia and bipolar disorder show aberrant MMN and ASSR compared to population-based controls.

**Methods:**

We will investigate MMN and 40 Hz ASSR in 15 year old adolescents (n ≈ 175) born to parents diagnosed with either SZ (FHR-SZ), BD (FHR-BD), or neither SZ or BD (population-based controls, PBC) using MEG. We will first perform sensor-level analyses and subsequently apply dynamic causal modeling (DCM) to investigate effective connectivity and make inferences about the underlying neurobiological mechanisms. We will investigate whether current psychopathology, cognitive status, and genetic risk for SZ and BD predict neurophysiological responses in the adolescents. Investigations are part of The Danish High Risk and Resilience Study - VIA (VIA15), a population-based longitudinal cohort study integrating social, psychological and biological risk factors and outcomes for SZ and BD.

**Results:**

Final results are expected in 2024

**Conclusions:**

The VIA15 study will allow for unprecedented insight into the neurobiological development of schizophrenia and bipolar disorder.

**Disclosure:**

No significant relationships.

